# Identification of thyroid hormone response genes in the remodeling of dorsal muscle during *Microhyla fissipes* metamorphosis

**DOI:** 10.3389/fendo.2023.1099130

**Published:** 2023-02-03

**Authors:** Lusha Liu, Qi Liu, Xue Zou, Qiheng Chen, Xungang Wang, Zexia Gao, Jianping Jiang

**Affiliations:** ^1^ Chengdu Institute of Biology, Chinese Academy of Sciences, Chengdu, China; ^2^ College of Fisheries, Huazhong Agricultural University, Wuhan, China; ^3^ University of Chinese Academy of Sciences, Beijing, China; ^4^ Northwest Institute of Plateau Biology, Chinese Academy of Sciences, Xining, China; ^5^ CAS Key Laboratory of Mountain Ecological Restoration and Bioresource Utilization & Ecological Restoration and Biodiversity Conservation Key Laboratory of Sichuan Province, Chengdu, China

**Keywords:** TH, metamorphosis, dorsal muscle remodeling, *Microhyla fissipes*, target gene, SMRT sequencing, Illumina sequencing

## Abstract

**Introduction:**

Extensive morphological, biochemical, and cellular changes occur during anuran metamorphosis, which is triggered by a single hormone, thyroid hormone (TH). The function of TH is mainly mediated through thyroid receptor (TR) by binding to the specific thyroid response elements (TREs) of direct response genes, in turn regulating the downstream genes in the cascade. The remodeling of dorsal skeletal muscle during anuran metamorphosis provides the perfect model to identify the immediate early and direct response genes that are important during apoptosis, proliferation, and differentiation of the muscle.

**Methods:**

In our current study, we performed Illumina sequencing combined with single-molecule real-time (SMRT) sequencing in the dorsal muscle of *Microhyla fissipes* after TH, cycloheximide (CHX), and TH_CHX treatment.

**Results and Discussion:**

We first identified 1,245 differentially expressed transcripts (DETs) after TH exposure, many of which were involved in DNA replication, protein processing in the endoplasmic reticulum, cell cycle, apoptosis, p53 signaling pathway, and protein digestion and absorption. In the comparison of the TH group vs. control group and TH_CHX group vs. CHX group overlapping gene, 39 upregulated and 6 downregulated genes were identified as the TH directly induced genes. Further analysis indicated that AGGTCAnnTnAGGTCA is the optimal target sequence of target genes for TR/RXR heterodimers in *M. fissipes*. Future investigations on the function and regulation of these genes and pathways should help to reveal the mechanisms governing amphibian dorsal muscle remodeling. These full-length and high-quality transcriptomes in this study also provide an important foundation for future studies in M. fissipes metamorphosis.

## Introduction

Amphibian metamorphosis is one of the most dramatic examples that undergo significant morphological, behavioral, physiological, biochemical, and cellular changes during post-embryonic development (1). Three transformative processes occur during metamorphosis: larval organ/tissue (e.g., tail and gill) resorption, adult organ/tissue (e.g., limb) development, and the remodeling of tadpole organ into the adult form (e.g., intestine, dorsal muscle, skin, and brain) (2–4). At the cellular level, there is a balance between cell death of larval organs/tissues and the proliferation and differentiation of adult progenitor/stem cells for the survival of individuals (5). These processes during metamorphosis were triggered by a single hormone: thyroid hormones (THs: T3 and T4). Inhibiting the synthesis of endogenous TH leads to the giant tadpole development ceasing at an early metamorphosis stage, but exogenous TH initiates precocious metamorphosis of premetamorphic tadpoles (6, 7). Metamorphosis can be easily manipulated by controlling the availability of TH to tadpoles, which is independent of any maternal influence (8). Thus, amphibian metamorphosis provides an ideal model to study postembryonic organ development and tissue remodeling (9). In addition, due to the dramatic morphological, biochemical, and cellular changes during amphibian metamorphosis, it also serves as a sensitive barometer to detect the effects of environmental chemical contaminants and environmental influence on TH action and endocrine function in vertebrates (10).

The function of TH is primarily mediated through thyroid hormone receptors (TRs), which are transcription factors (TFs) and are members of a large superfamily of nuclear receptors (11). TR mainly functions as a heterodimer with the 9-*cis*-retinoic acid receptor (RXR), which binds to the promoter regions (known as thyroid response elements (TREs)) of direct response genes to regulate transcription (12). Then, the products of these direct response genes affect downstream genes and pathways in the cascade and then lead to extensive morphological and physiological remodeling of tissues. This TRE contains a hexameric nucleotide (AGGTCA) direct repeat spaced by four bases (DR4) (13). A dual-function model has been proposed to describe the role of TR during metamorphosis. When TH is absent, the TR/RXR heterodimers recruit co-repressors to repress the expression of direct target genes (9, 14). Once they bind to TH, a conformational change in the TR induces it to release the co-repressor complex and to recruit the co-activator complex to activate the expression of downstream genes, leading to metamorphic changes (8).

Each tissue of the tadpole responds to TH at different levels, ranging from altered gene expression, cell fate, and morphogenesis to tissue restructuring (1). In addition to the degeneration of the tail and development of the limb, the dorsal muscle (also named trunk muscle) transforms from larval-type to adult-type during anuran metamorphosis (15). Of interest, two distinct types of myoblasts and myotubes exist in the dorsal region of the developing tadpole. Instead of the hypothesis “direct conversion of larval to adult type muscle”, studies revealed that TH induces cell death in the larval types and proliferation in adult types, called the “replacement model” (16). Adult-type myotubes are formed with new myogenesis at the dorsomedial part in trunk muscles and expand gradually from dorsal to ventral with an anteroposterior gradient (17). On the molecular regulation level, *MyoG* transcripts are accumulated in a few small, secondary myofibers expressing the adult MyHC, while they are not expressed in primary myofibers expressing the larval MyHC (18, 19). Furthermore, Hedgehog (Hh) signaling pathway plays a crucial role in adult myoblast differentiation (19, 20). All related work is restricted to the model organisms *Xenopus laevis* and *Xenopus tropicalis*, which are only representatives of Mesobatrachia. Therefore, it remains to investigate whether these findings in dorsal muscle remodeling are conserved in other anuran species. *Microhyla fissipes*, from the family of Microhylidae belonging to the Neobatrachia, is a small-sized and widely distributed anuran with a number of advantages for developmental and genetic studies, such as transparent tadpoles, large egg size, and diploid (9). Compared to *Xenopus*, *M. fissipes* develops faster and changes from an aquatic tadpole to a terrestrial frog during metamorphosis, which is closer to the postembryonic development in mammals (9, 21). Furthermore, we have previously found differentially expressed genes and miRNAs during *M. fissipes* metamorphosis, especially in tail resorption (8, 22, 23). However, the global gene expression changes and TH directly induced genes underlying dorsal muscle remodeling in *M. fissipes* are still unknown and elusive.

Here, we take advantage of Illumina RNA sequencing (RNA-seq) in combination with single-molecule real-time (SMRT) sequencing for transcriptome assembly on *M. fissipes* dorsal muscle exposed to 0.1× MMR alone, TH, protein synthesis inhibitors (cycloheximide (CHX)), and TH with CHX (TH_CHX) to systematically identify regulation profiles in dorsal muscle remolding. Gene Ontology (GO) and Kyoto Encyclopedia of Genes and Genomes (KEGG) analyses showed that these genes are enriched in categories/pathways vital for the earliest steps of cellular transformations during *M. fissipes* metamorphosis. In addition, based on the more stringent criterion, the overlapped regulated genes between TH *vs.* control and TH_CHX *vs.* CHX were identified as the TH direct response gene, which plays important roles in propagating the effects of TH in regulating dorsal muscle during metamorphosis. This study revealed a number of signaling transduction pathways regulated by TH as the first step toward inducing metamorphosis and identified many direct TR target genes. These genes also provide the potential biomarker of endocrine disruption and genetic data for other subsequent studies of *M. fissipes*. These results should help to elucidate the mechanisms governing amphibian dorsal muscle remodeling.

## Materials and methods

### Experimental samples

*M. fissipes* tadpoles were obtained from one pair of frogs by artificial spawning and maintained as previously described (8). Tadpoles at stage 28 (prometamorphosis, 4 days old) were treated as four groups using 0.1× Marc’s Modified Ringer Solution (MMR) alone, 100 nM of 3,5,3′-triiodo-l-thyronine (TH, Sigma Chemical Co., St. Louis, MO, USA), 20 μg/ml of CHX (Cell Signaling Technology, Boston, MA, USA), which inhibits protein synthesis *in vivo*, and 100 nM of TH with 20 μg/ml of CHX (TH_CHX). Each treatment included 15 tadpoles and had 4 replicates. Tadpoles maintained in 0.1× MMR alone were set as the control group. For tadpoles treated with both TH and CHX (TH_CHX group), the tadpoles were treated with CHX for 1 h before TH addition, and then these tadpoles were treated with CHX and TH for 14 h. The tadpoles were exposed to TH for 14 h (TH group) or CHX for 15 h (CHX group). Tadpoles in one group were randomly collected and anesthetized with 0.01% MS222. The dorsal part without the skin of tadpoles was dissected, and then the dorsal side muscle around the spine was dissected, immediately immersed in liquid nitrogen, and stored at −80°C until the RNA was extracted. The other five dorsal parts of tadpoles were collected for TUNEL assay. All animal care and treatments were performed as approved by the Experimental Animal Use Ethics Committee of the Chengdu Institute of Biology (Permit Number: 2021010).

### Illumina RNA sequencing

The total RNA of each sample was extracted using the TRIZOL Kit (Invitrogen, Carlsbad, CA, USA) according to the manufacturer’s instructions. The concentration and the integrity of RNA were checked using Agilent 5400 Bioanalyzer (Agilent Technologies, Palo Alto, CA, USA) and Nanodrop Spectrophotometer (IMPLEN, Westlake Village, CA, USA). After quality control, 16 libraries were sequenced on the Illumina NovaSeq 6000 platform (Illumina Inc., San Diego, CA, USA) with 150-nt paired-end reads by NovoGene (Beijing, China). The transcriptome data of all the samples were uploaded to the Genome Sequence Archive in National Genomics Data Center (https://ngdc.cncb.ac.cn/gsa/), with the accession number CRA008592.

### Data analysis of PacBio SMRT sequencing

The detailed samples and method for single-molecule RNA real-time sequencing were described previously (22). Full-length transcripts were obtained by using the SMRTlink 5.0 software. Then, full-length transcripts were corrected using the Illumina RNA-seq reads by LoRDEC (24). The proofread-corrected sequences after removal of the redundant sequences by CD-HIT were annotated by querying databases NR (Non-Redundant Protein Sequence Database), NT (Nucleotide Sequence Database), Swiss-Prot, KOG (Clusters of orthologous groups for eukaryotic complete genomes), Pfam, GO, and KEGG (25).

Transcription factors are proteins that bind to specific nucleotide sequences upstream of a gene and regulate the binding of RNA polymerase to DNA templates, thereby regulating gene transcription. Animal transcription factors were predicted using the animal TFDB 2.0 database. We used CNCI, CPC, Pfam-scan, and PLEK four tools to predict the coding potential of transcripts. Transcripts without coding potential were our candidate set of lncRNAs.

### Analysis of differentially expressed transcripts

To detect different gene expressions after treatment in the dorsal muscle of *M. fissipes*, the final corrected clean reads from SMRT-seq were used as the reference sequences. After the removal of the reads with adapters-only reads, low-quality reads, and reads with unknown nucleotides, clean reads were mapped against the reference sequences above using Bowtie. Then, gene expression levels for each sample were estimated using RNA-seq by expectation–maximization (RSEM) (26). To evaluate the gene expression in 16 samples, the number of unique-match reads was calculated and then normalized to FPKM (fragments per kilobase of transcript per million mapped reads). Subsequently, the DESeq package (1.18.0) was used to identify differentially expressed transcripts (DETs) with |log_2_FC| ≥ 1, padj value < 0.05 after adjustment for the false discovery rate (FDR). Then, DETs were analyzed using GO enrichment and KEGG pathway enrichment by the GOseqR package (27) and the KOBAS software (28), respectively. *q* < 0.05 was significantly enriched. For DETs related to cell proliferation and cell apoptosis, log_2_FPKM values of genes were used for heatmap generation with the pheatmap package in R (Version 3.0.3). In order to identify the direct target gene of TH, DETs between TH and control were compared with DETs between TH_CHX and CHX. Overlapping DETs in these two comparisons were identified as the TH direct response gene.

### Terminal deoxynucleotidyl transferase-mediated deoxyuridine triphosphate nick end labeling (TUNEL) staining

Cell apoptosis in dorsal muscle was detected by a DAB (SA-HRP) TUNEL Cell Apoptosis Detection Kit (G1507, Servicebio, Wuhan, China) according to the manufacturer’s protocol. Cross sections were scanned and observed using a Pannoramic MIDI Scanner (3DHISTECH Kft., Budapest, Hungary) and Pannoramic Viewer software (3DHISTECH, Budapest, Hungary). The percentage of apoptotic cells was calculated in the dorsal muscle. The percentage of apoptotic cells = number of positive apoptosis cells/(number of positive apoptosis cells + number of negative apoptosis cells) × 100%. The positive apoptosis cells developed by the DAB reagent have a brown-yellow nucleus, and negative apoptosis cells were stained only blue. All quantitative results were presented as the mean ± SEM, and a value of *p* < 0.001 was considered to be statistically highly significant.

### Validation of putative TH directly regulated genes by qRT-PCR

Twelve genes were randomly selected from the 46 putative target genes for qPCR analysis to validate the results from our RNA sequencing. Three samples from the control group, CHX group, TH group, and TH_CHX group were used for qRT-PCR, and *rpl37* gene was used as an internal control. PCR primers used for qRT-PCR amplification are listed in **Table 1**. Total RNA was isolated from each sample using TRIzol reagent (TaKaRa, Dalian, China), while cDNAs were synthesized using HiScript™ Q RT SuperMix for qPCR (Vazyme, Nanjing, China) according to the manufacturer’s protocol. qPCRs were performed on a QuantStudio™ 6 Flex qRT-PCR system (ABI, Foster City, CA, USA) using Hieff^®^ qPCR SYBR Green Master Mix (Yeasen, Shanghai, China). The relative amount of expression levels of different genes was calculated using the 2^−ΔΔct^ method. The level of significance was determined by one-way analysis of variance (ANOVA) with SPSS Statistics 13.0. All quantitative results were presented as the mean ± SEM, and a value of *p* < 0.05 was statistically significant, while p < 0.0001 was highly significant.

**Table 1:** Primers used in gene expression analysis by quantitative real-time PCR.

Gene	Forward primer	Reverse primer
*TMLHE*	CACCTTATTGGTGGACGGCT	GCTGGGTCCGATGTTCTCAA
*PFAS*	GCGGGAGGGACCCAAATATC	TCTCCCGGTCTCCGTTACTT
*TH/bZIP*	TGCAAAACGTTCGAGGGAGA	GGATCACGTACCAGGCCAAA
*KLF9*	TTCGGTGTCCCCTTTGTGAG	GCTGAACTGGACCTCTTGGA
*DUSP9*	CCAACACGGTTAGGAGTCCA	TTCTGCCTGGAACTTGCTGA
*SPG20*	TGACACCAGACGGACAAGTG	GCCAATCACACACCTGGAGA
*CCN4*	CCGCATCTCCAACAACAACG	CTAGGCACTTCTTACCCGGC
*CYP2K4*	CGTTCCTGGCAAAGCAACAA	TTCCATTCCGGCACCAAAGA
*TRβ*	TGGCCAAAACTGCTGATGAA	CGTGGCAGGCTCCAA
*BGLAP*	AGCTGAACCCAGACTGTGATG	GACACGAGGAGCTAGGCAGA
*Sox4*	GCAGAACATTGAGCCACACA	GTACAAGCTCCCGAGCAAGA
*CMKLR1*	TCCTCGGTTGGGAATGCAAA	CTCCAGCGGCTTTTTATGGC
*rpl37*	CCAAAAAGCGCAACAACCA	TTGCGAATCTGACGGACTTG

### TRE prediction in validated target genes

For TRE prediction, a 5,000-bp sequence upstream of the putative transcriptional start site of these 12 target genes validated by qPCR was used. To search regions for TRE, the pattern (AGGTCANNNNAGGTCA) allowed with four mismatches was used by IdentityX in the Mac App Store.

## Results

### SMRT and Illumina sequencing of *M. fissipes*

SMRT and Illumina sequencing approaches were combined to generate high-quality reference transcriptome and comprehensive transcriptional profiles among different treatment groups. These PacBio SMRT Bell libraries (1–2, 2–3, and 3–6 kb) were sequenced with 10 SMRT cells, yielding 215,301,817 nucleotide sequences. In total, 602,931 reads of the insert were generated, among which 385,666 were identified as full-length non-chimeric (FLNC) reads. As SMRT sequencing has a relatively high error rate, 104.94-Gb clean reads from Illumina RNA sequencing of 16 samples were used to further correct SMRT transcripts. After error correction and redundant transcript removal, 63,041 non-redundant transcripts (0–1 kb (20), 1–2 kb (24,307), 2–3 kb (32,730), and >3 kb (5,984)) were generated as the reference transcriptome of *M. fissipes* in this study. The lengths of these assembled transcripts ranged from 430 to 15,740 bp with an average length of 2,222 bp and an N50 of 2,299 bp (**Table S1**), which is longer than SMART sequencing analysis before with an average length of 1,599 bp and an N50 of 1,956 bp (22). For functional annotation, 42,499 transcripts were annotated in the NR database, 33,502 in NT, 24,577 in KO, 36,070 in Swiss-Prot, 36,460 in Pfam, 36,454 in GO, and 16,492 in KOG. Overall, 51,407 (81.54%) transcripts were annotated in at least one of the databases (**Table S1**), compared to 78% annotated transcripts from SMART sequencing analysis before (22). The N90 of SMRT sequencing data is 1,495 bp compared to 446 bp for *de novo* transcripts. For Illumina sequencing, 135,627 transcripts were obtained with an average length of 1,049 bp and an N50 of 1,539 bp (**Table S2**), which were much shorter than our SMRT sequencing data. Furthermore, *de novo* transcripts in the range of 300–500 bp account for the most at 37.08% (50,295), while only 0.0016% of that is in SMRT sequencing data (**Tables S1**, **S2**), while the annotation rate of *de novo* transcripts 43.33% in at least one database was much lower than that of SMRT transcripts (**Table S2**). These data indicated that transcripts obtained from SMRT sequencing in this study were highly improved compared to SMRT sequencing analysis before and *de novo* RNA-seq data.

Furthermore, 29,900, 31,477, 22,962, and 35,937 lncRNAs were predicted by CPC, CNCI, PLEK, and Pfam analysis, respectively. In total, 16,234 lncRNAs were predicted by these four methods (**Figure S1A**). In addition, 2,265 TFs were identified in this study, and zf-C2H2 was the most enriched with 1,373 TFs, followed by the ZBTB with 137 TFs, Homeobox with 112 TFs, and TF_bZIP with 75 TFs (**Figure S1B**).

### Identification of DETs in dorsal muscle during TH-induced metamorphosis

**Figure 1:**
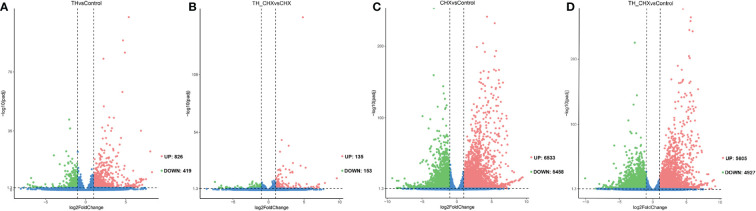
Volcano plots of differentially expressed transcripts (DETs) in the dorsal muscle for four pairwise comparisons. **(A)** TH *vs.* control. **(B)** TH_CHX *vs.* CHX. **(C)** CHX *vs.* control. **(D)** TH_CHX *vs.* control. The x-axis displays the log2 fold change value of the DETs, and the y-axis corresponds to the −log10 padj value of the DETs, with 0.05 set as the significance level cutoff. Upregulated DETs are shown in red; downregulated DETs are in green; non-significant DETs are presented in blue. TH, thyroid hormone; CHX, cycloheximide.

To investigate the molecular mechanisms underlying TH-induced dorsal muscle remodeling, we performed a global analysis of gene expression by Illumina RNA-seq on dorsal muscles after TH treatment. By comparing the dorsal muscle transcriptomes of the TH group and control group, we identified 1,245 DETs, out of which 826 were increased after TH treatment and 419 were downregulated (|log2FC| ≥ 1, padj value < 0.05) (**Figure 1A**; **Table S3**).

The genes regulated by TH alone likely included immediate early, direct response, and late response genes, with the regulation of the latter sensitive to CHX. When compared with the CHX group, 135 genes were upregulated and 153 genes were downregulated by TH in the presence of CHX (**Figure 1B**; **Table S3**).

Furthermore, exposure to CHX and TH_CHX significantly modulated the expression of 11,991, and 10,532 genes in the dorsal muscle tissue compared to the control tadpoles, respectively (**Figures 1C, D**; **Table S3**). Of these DETs, 6,533 and 5,605 were upregulated relative to control while 5,458 and 4,927 were downregulated by each treatment. The number of DETs in *M. fissipes* after different treatments was much more than in *X. laevis* with the same treatment by microarray (11). These data indicated that RNA-seq could detect new genes and was more sensitive.

To compare the global pattern of transcriptional response induced by TH, CHX, and TH_CHX, hierarchical cluster analysis of the all identified DETs across four groups demonstrated that the TH group and control group DETs clustered together and that the TH_CHX group clustered with the CHX group (**Figure S2**).

### Functional classification of the DETs identified during TH-induced dorsal remodeling

**Figure 2:**
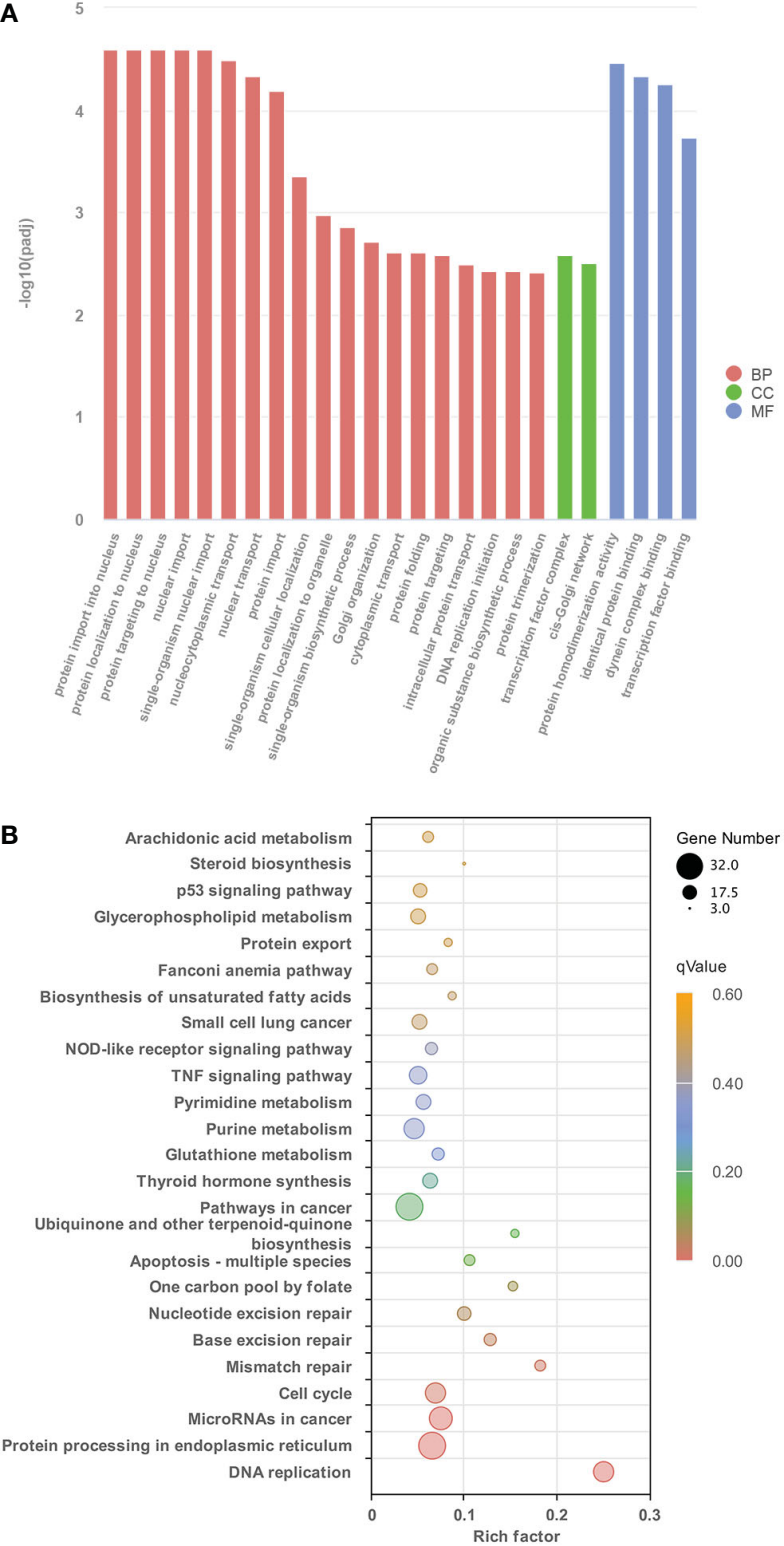
The characteristics of GO terms and KEGG pathways significantly enriched between control group and TH treatment group. **(A)** The top 25 GO terms enriched in DETs identified from control group and TH treatment group (*p* < 0.05) **(B)** Significantly enriched KEGG pathways of DETs between control group and TH treatment group (*p* < 0.05). GO, Gene Ontology; KEGG, Kyoto Encyclopedia of Genes and Genomes; TH, thyroid hormone; DETs, differentially expressed transcripts.

To understand the global gene functional categories and biological pathways during dorsal muscle remodeling, enrichment analysis was performed on the 1,245 TH-induced genes between the TH and control groups. These analyses revealed 128 significantly enriched GO categories (*q* < 0.05) (**Table S4**; **Figure 2A**) in three main GO categories: biological process (BP), cellular component (CC), and molecular function (MF). The significantly enriched GO terms and their categories are listed in **Table S4**. Most significantly enriched GO terms were strongly associated with cell division such as protein import into the nucleus, protein complex assembly, transcription factor complex, and DNA replication initiation. Six KEGG pathways including “Protein processing in endoplasmic reticulum”, “MicroRNAs in cancer”, “Cell cycle”, “Mismatch repair”, and “Base excision repair” were significantly enriched in these DETs (*q* < 0.05) (**Figure 2B**). Furthermore, another 19 significantly enriched KEGG pathways were found based on the less stringent criterion with a value of *p* < 0.05 (**Figure 2B**; **Table S5**).

Some of the GO categories and KEGG pathways enriched in TH-induced genes (**Figure 2**) were not enriched in TH_CHX-regulated genes (**Figures S3**, **S4**), such as DNA replication genes and the protein process in the nucleus.

### Coordinated regulation of gene categories related to dorsal remodeling during TH-induced metamorphosis

**Figure 3:**
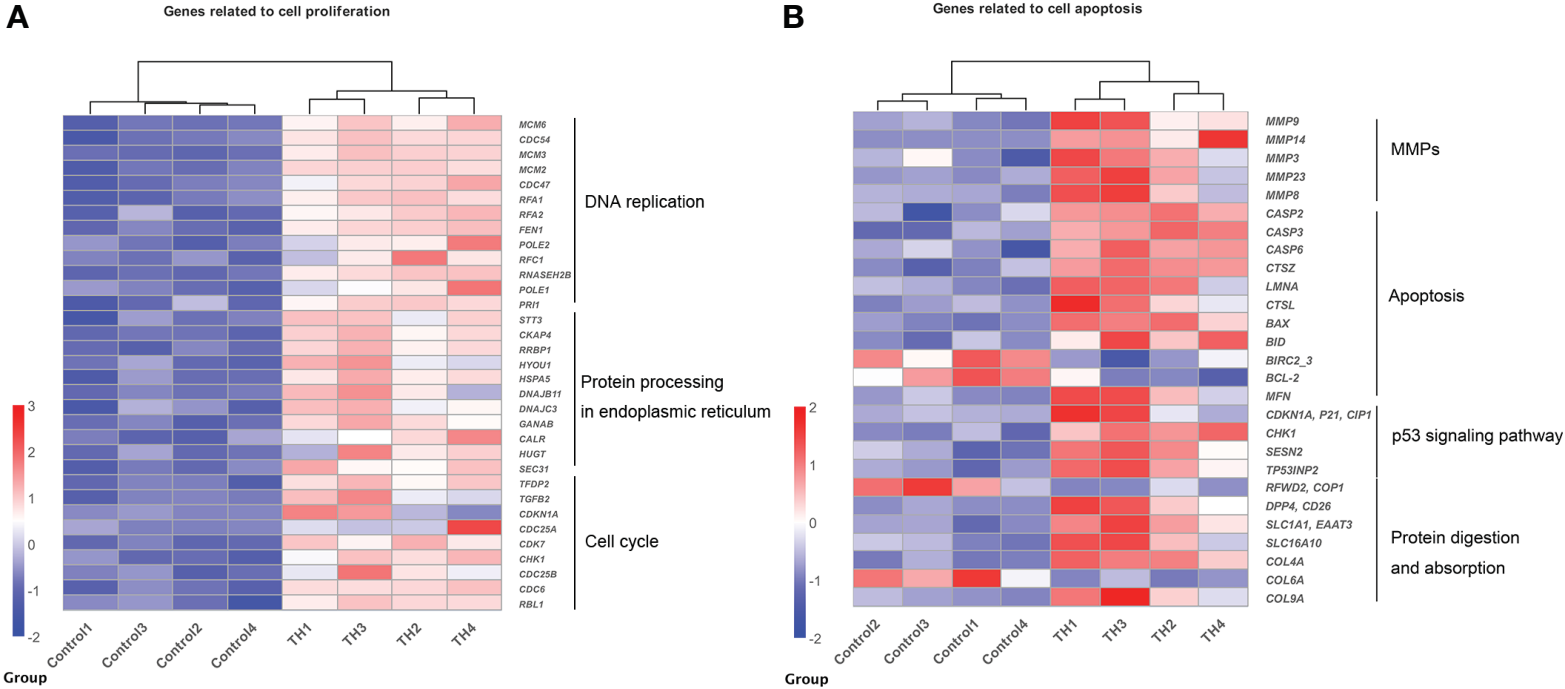
Heatmaps showing coordinated regulation of DETs in different cell processes between control group and TH treatment group. **(A)** A heatmap of all DETs related to cell proliferation, which includes DNA replication, protein process in endoplasmic reticulum, and cell cycle KEGG pathway. All genes were upregulated after TH exposure. **(B)** A heatmap of DETs related to cell apoptosis, which includes MMPs, apoptosis, p53 signaling pathway, and protein digestion and absorption KEGG pathway. Nearly all were upregulated after TH exposure except *BIRC2_2*, *BCL-2*, *COP1*, and *COL6A*. The intensity of color indicates relative expression levels. Red to blue corresponds to high to low levels of expression. DETs, differentially expressed transcripts; TH, thyroid hormone; KEGG, Kyoto Encyclopedia of Genes and Genomes; MMPs, matrix metalloproteinases.

Genes enriched in these pathways/processes were further compared and integrated into the cell proliferation and cell apoptosis consensus module manually. The cell proliferation module was composed of core components of DNA replication, protein processing in the endoplasmic reticulum, and cell cycle (**Figure 3A**). In contrast, the cell apoptosis module was composed of core components of matrix metalloproteinases (MMPs), apoptosis, p53 signaling pathway, and protein digestion and absorption (**Figure 3B**).

### Apoptosis of *M. fissipes* dorsal muscle after TH treatment

**Figure 4:**
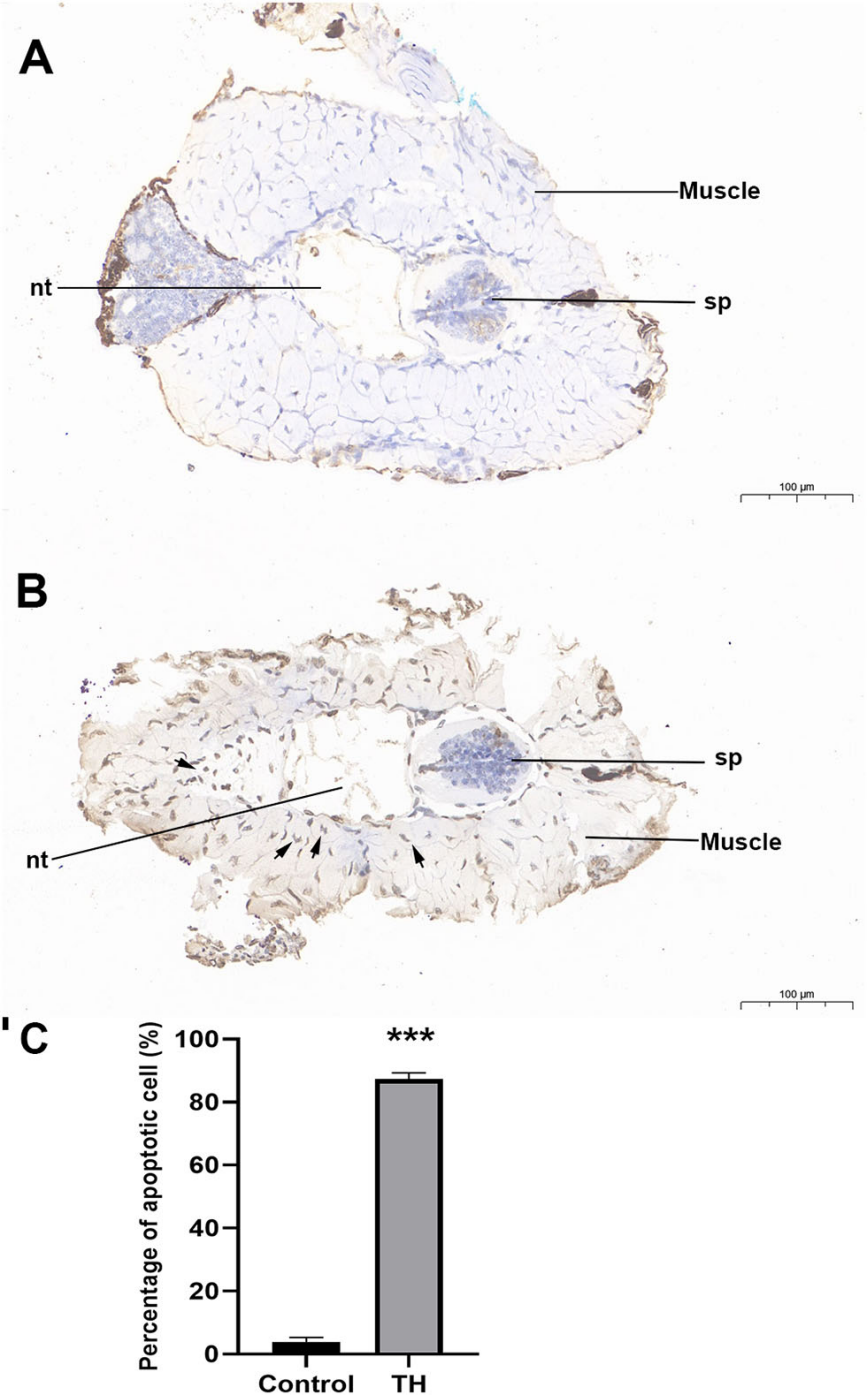
Representative images of TUNEL staining of dorsal of *Microhyla fissipes* in control group **(A)** and TH treatment group **(B)** (magnification, ×15; scale bar, 100 μm). **(C)** Apoptosis cells rate in the dorsal muscle of control and TH exposure. Nuclei stained with hematoxylin are blue, while the positive apoptosis cells developed by DAB reagent have a brown-yellow nucleus. Arrow, TUNEL-positive cell; nt, notochord; sp, spinal cord; TH, thyroid hormone. *** indicate significant differences between samples at p < 0.001 based on ANOVA analysis.

During TH-induced dorsal muscle remodeling, cell apoptosis has been enriched in the KEGG enrichment analysis. We performed TUNEL staining for *in situ* detection of cellular apoptosis after TH treatment. As shown in **Figures 4A, B**, only a blue nucleus was observed in the control group, while the brown-yellow nucleus, which indicated the apoptosis-positive cells, was obviously increased by TH treatment. Furthermore, quantitative analysis showed that the apoptosis cell rate was highly significantly increased after TH exposure (**Figure 4C**).

### Identification of the direct response genes of TH in *M. fissipes*

**Figure 5:**
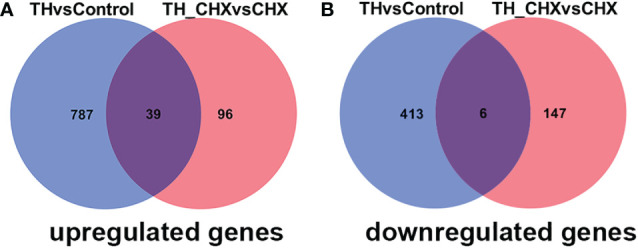
Venn diagrams illustrating the overlap in DETs between TH *vs.* control and TH_CHX *vs.* CHX. The numbers in each circle (blue circle, TH *vs.* control; red circle, TH_CHX *vs.* CHX) indicate the total number of different genes in each comparison group, and the number in the overlapping areas is the number of shared genes between two comparison groups. **(A)** A total of 826 genes were upregulated by TH alone (TH group compared with control group), whereas 135 were upregulated by TH in the presence of CHX (TH_CHX group compared with CHX group). Among them, 39 genes were commonly upregulated by TH or TH_CHX. **(B)** A total of 419 genes were downregulated by TH alone (TH group compared with control group), whereas 153 were downregulated by TH in the presence of CHX (TH_CHX group compared with CHX group). Among them, six genes were commonly downregulated by TH or TH_CHX. DETs, differentially expressed transcripts; TH, thyroid hormone; CHX, cycloheximide.

The genes regulated by TH alone included immediate early, direct response, and later response genes. The genes regulated by TH_CHX included immediate early, direct TH response, and CHX response genes. After comparisons, 826 upregulated genes and 419 downregulated genes were detected between the TH group and control group, while 135 upregulated genes and 153 downregulated genes were detected between the TH_CHX group and CHX group. Based on the more stringent criterion, the overlapped regulated genes between these two comparisons were identified as the TH direct response gene (**Figures 5A, B**). Thus, 39 genes commonly upregulated and 6 genes commonly downregulated by TH and TH_CHX treated were the TH target genes (**Table 2**). *TRβ* and *TH/bZIP* were the widely known direct upregulated genes in *Xenopus*, while *SOX4*, *KLF9*, and *MGP* were directly regulated by TH/TR/RXR in *Xenopus* or rats. The other genes, such as *PFASF*, *DUSP9*, *CCN4*, *CYP2K*, *SPG20*, *SOX4*, *BGLAP*, CMKLR, and *TMLHE*, were newly identified as the direct upregulated genes in dorsal muscle remodeling. These 45 genes directly responding to TH also may represent candidate biomarkers assessing the effects of environmental chemical contaminants on TH signaling in vertebrates.

**Table 2:** List of direct TH-regulated genes identified by RNA-seq.

Unigene ID	Annotation description	Symbol	TH *vs.* control		TH_CHX *vs.* CHX	
			log_2_FC	*q* value	log_2_FC	*q* value
1-2.c12061_1_1454	Mucin-19	*MUC19*	1.836	2.24E−06	1.1971	7.78E−05
1-2.c14617_5_1744	Thyroid hormone receptor beta	*TRβ*	4.96	0.000225	2.75	0.009164
1-2.c18325_5_1432	Cellular communication network factor 4	*CCN4*	2.8591	2.39E−10	1.4166	7.07E−08
1-2.c29621_1_1669	Phosphoribosylformylglycinamidine synthase	*PFAS*	1.4898	0.00025478	2.9301	1.42E−19
1-2.c40374_1_1646	Cytochrome P450 2K6	*CYP2K6*	3.4304	5.71E−08	1.8504	0.00055797
1-2.c48220_1_1626	Thyroid hormone-induced bzip protein	*TH/BZIP*	6.8902	7.79E−36	4.5078	2.34E−23
1-2.c49674_1_1423	SRY-box 4	*SOX4*	1.6237	5.45E−11	1.0687	4.52E−12
1-2.c51085_1_1665	Aldo-keto reductase family 1 member C1 homolog	*AKR1C1*	3.3202	4.03E−07	2.6031	7.47E−05
1-2.c5151_6_1369	BTB/POZ domain-containing protein KCTD1	*KCTD1*	4.0778	1.74E−14	3.4215	5.18E−26
2-3.c10814_9_2303	HIG1 domain family member 1C	*HIGD1C*	2.0233	4.56E−17	1.5861	6.19E−20
2-3.c13088_1_2345	*Xenopus tropicalis* clone ISB-376E5		4.5116	1.77E−09	2.8316	8.01E−07
2-3.c14039_1_2467	Cyclic AMP-responsive element-binding protein 3-like protein	*CREB3L2*	1.1192	0.011793	1.0293	0.020369
2-3.c15607_1_2004	Matrix Gla protein	*MGP*	2.2257	1.89E−12	1.4194	2.14E−06
2-3.c22743_1_2511	Domain of unknown function		1.0446	4.72E−05	1.1645	9.09E−07
2-3.c25947_1_2264	Integrase core domain		3.1629	8.27E−36	2.0716	3.45E−23
2-3.c28146_1_1987	High mobility group AT-hook 2	*HMGA2*	2.2612	2.49E−50	1.5387	8.26E−20
2-3.c29527_1_2235	Nuclear factor I B	*NFIB*	2.0185	1.80E−20	1.1458	0.00079244
2-3.c33125_1_2127	Prolactin-releasing peptide		2.2236	7.57E−16	1.2502	9.35E−09
2-3.c35212_1_1923	30S ribosomal protein Thx		6.2648	5.50E−06	1.8219	0.019126
2-3.c3870_2_2171	High mobility group protein HMGI-C isoform X1	*HMGI-C*	3.2162	5.44E−08	1.4332	0.0063144
2-3.c40223_1_2454	Primase zinc finger		1.9637	0.0070026	2.5371	1.71E−05
2-3.c41889_1_2446	Phospholysine phosphohistidine inorganic pyrophosphate phosphatase-like isoform X1		8.053	1.30E−23	2.8319	3.54E−42
2-3.c46259_1_2693	LINE-1 retrotransposable element ORF2 protein	*POL*	3.5154	1.61E−43	2.4839	1.25E−12
2-3.c46777_1_2141	Cytochrome P450 2K4-like	*CYP2K4*	3.7652	3.02E−14	5.3556	3.21E−06
2-3.c49466_1_2588	Bone gamma-carboxyglutamate protein, osteocalcin	*BGLAP*	4.58	2.93E−05	3.85	4.32E−05
2-3.c49620_1_2429	A-agglutinin-binding subunit Aga2		2.5667	5.68E−17	1.9347	4.84E−09
2-3.c49900_1_2283	Chemokine-like receptor 1	*CMKLR1*	2.8313	0.00050004	2.1867	0.0024274
2-3.c54695_1_2043	Herpesvirus latent membrane protein 1	*LMP1*	6.7631	3.54E−07	5.2217	1.70E−21
2-3.c54887_1_2006	Endosialin-like		5.3677	3.01E−103	4.8179	1.81E−162
2-3.c56689_1_2256	Spastic paraplegia 20	*SPG20*	2.3087	1.16E−05	1.1046	0.01316
2-3.c59493_1_2681	Interferon-induced transmembrane protein		2.0567	3.01E−24	1.4189	6.19E−15
2-3.c59735_1_1918	Dual specificity protein phosphatase 9	*DUSP9*	1.5732	0.042605	1.0431	0.028172
2-3.c60998_2_1874	Serine protease	*HTRA1*	2.1584	1.34E−12	1.3218	8.01E−05
2-3.c8238_1_2510	*Streptopelia turtur* genome assembly, chromosome: Z		4.5727	7.72E−59	1.3346	2.68E−19
2-3.c8249_11_2151	Trimethyllysine dioxygenase	*TMLHE*	1.3585	3.27E−09	1.3164	2.25E−06
3-6.c13750_1_2763	*Nanorana parkeri* uncharacterized LOC108785573 (LOC108785573)		4.7756	3.73E−13	3.3546	1.11E−17
3-6.c5228_1_3273	Neuronal regeneration related protein	*NREP*	4.6538	1.94E−89	1.6176	1.72E−36
3-6.c5722_1_2767	Hematopoietic prostaglandin D synthase	*HPGDS*	2.7747	4.67E−39	1.0969	0.0077565
3-6.c6057_1_8371	Kruppel-like factor 9	*KLF9*	3.8933	1.95E−16	1.4259	4.52E−07
1-2.c9181_5_1822	Type II keratin, basic	*KRT2*	−1.1652	0.001047	−1.5553	0.001456
1-2.c9370_4_1536	Type I keratin, acidic	*KRT1*	−2.2548	7.56E−12	−2.271	0.02986
2-3.c2430_14_2348	Calpain-9	*CAPN9*	−1.3212	3.15E−05	−1.8402	0.004251
2-3.c46963_1_2178	Actin beta/gamma 1	*ACTB_G1*	−3.3006	0.009307	−2.539	0.042791
3-6.c14529_1_2954	–		−1.047	0.030231	−3.9806	0.021854
3-6.c17071_1_3282	Endothelin receptor type B	*EDNRB*	−1.8683	7.62E−05	−1.1902	0.022074

TH, thyroid hormone; CHX, cycloheximide.

### Confirmation of the TH direct upregulated genes by qPCR

**Figure 6:**
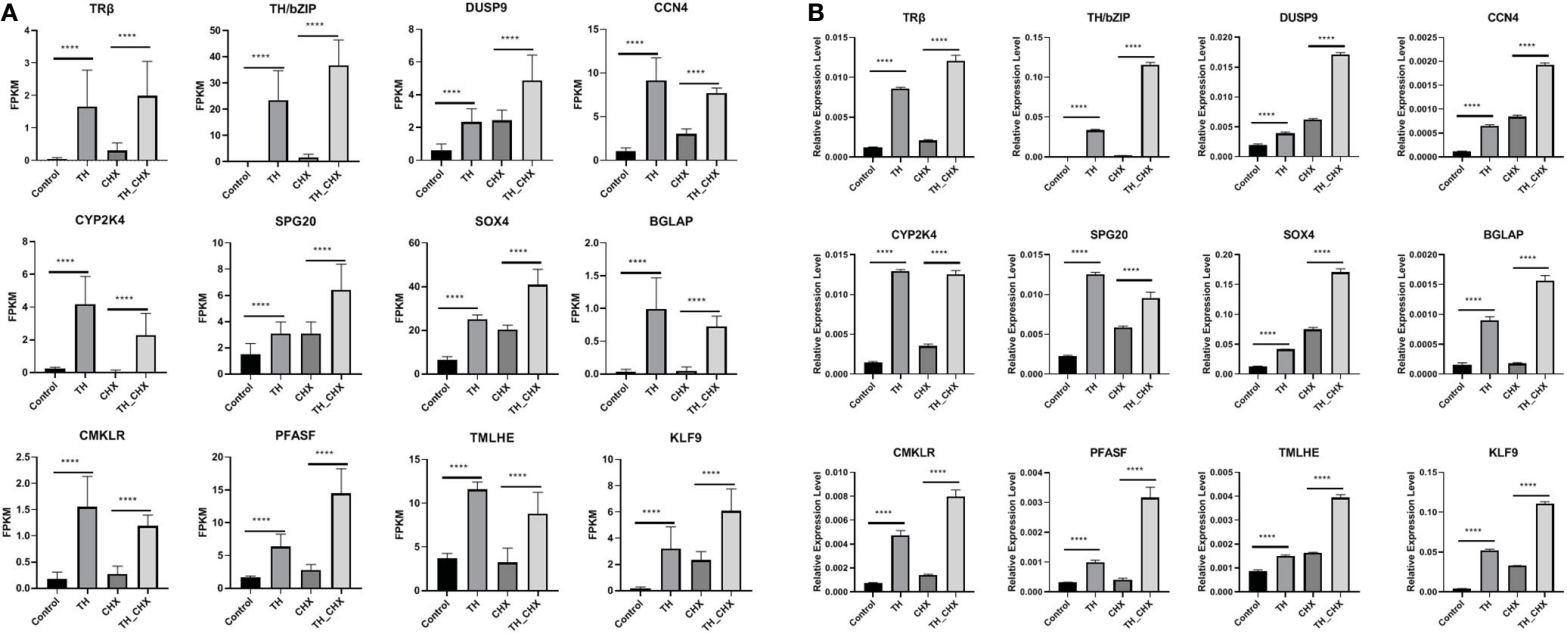
Expression patterns of selected genes in four groups (control, CHX, TH, and TH_CHX) determined by RNA-seq **(A)** and qPCR **(B)**. **(A)** In RNA-seq data, the vertical axis represents expression levels (FPKM value), and the horizontal axis represents four groups. The expression level for each sample is the means ± SD of four replicates. **(B)** For qPCR, the data are means ± SD from three independent replicates, and **** indicate highly significant differences between samples at *p* < 0.0001 based on ANOVA. CHX, cycloheximide; TH, thyroid hormone.

To validate the TH directly regulated gene by RNA-seq analysis, 12 genes (*TRβ*, *TH/bZIP*, *PFASF*, *DUSP9*, *CCN4*, *CYP2K*, *SPG20*, *SOX4*, *BGLAP*, *CMKLR*, *TMLHE*, and *KLF9*) with highly significant expression changes after TH and TH_CHX treatment were randomly selected for qRT-PCR analysis. The expression patterns obtained by qRT-PCR for the control *vs.* TH and CHX *vs.* TH_CHX were highly consistent with the sequencing results (**Figures 6A, B**; **Table 2**).

### Bioinformatics analysis of putative TREs in the validated target genes

**Figure 7:**
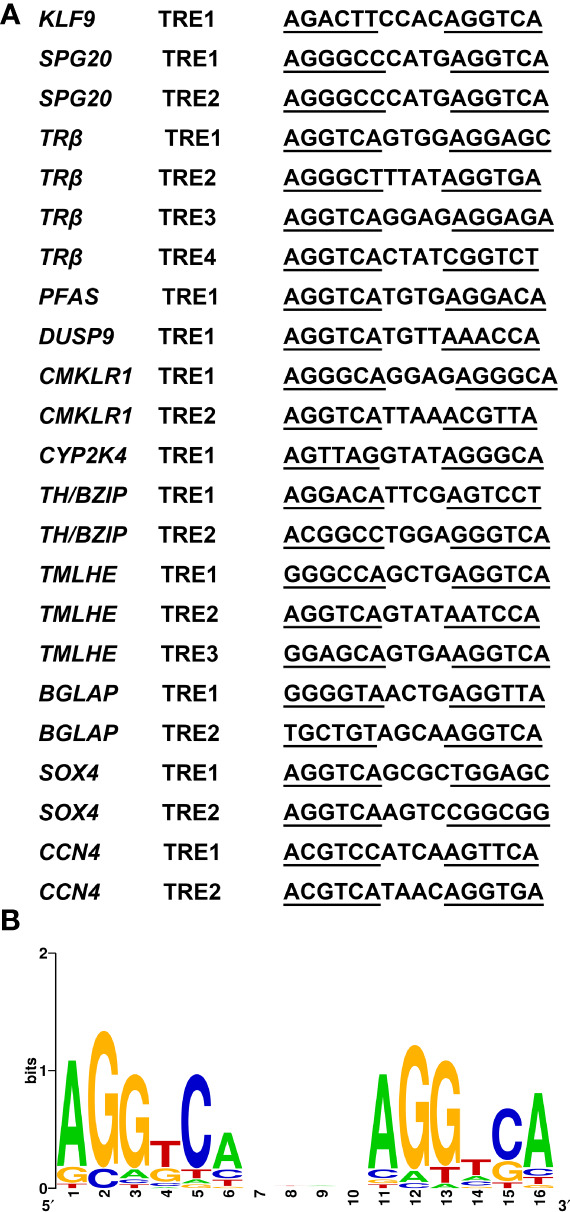
A consensus TRE predicted in *Microhyla fissipes* TR target genes. **(A)** Sequences of the 23 predicted TREs in *M. fissipes*. **(B)** Graphic representation of the consensus TRE generated from the 23 TREs above. The horizontal axis represents the positions of residues in the TRE, and vertical axis represents the relative frequencies of the four bases at each position as bits. TRE, thyroid response elements; TR, thyroid receptor.

Thus, to investigate whether the CHX-resistant T3 response genes were direct TR target genes, we needed to determine whether the genes contained functional TREs. This search yields TRE element in all promoters of TH direct upregulated genes identified. By using WebLogo (29), we generated a consensus TRE for *M. fissipes* TR target genes (**Figures 7A, B**) from the 23 newly identified TREs. The results from this rather larger set of biochemically characterized naturally occurring TREs indicate that AGGTCAnnTnAGGTCA is the optimal target sequence of endogenous target genes for TR/RXR heterodimers in *M. fissipes*, consistent with previous findings from *Xenopus*.

## Discussion

Amphibian metamorphosis has long been used as a model to study the actions of TH and molecular mechanisms underlying organ development and tissue remodelings, such as limb development, skin remodeling, tail resorption, intestine remodeling, brain remodeling, and dorsal muscle remodeling (8). Dorsal muscle remodeling is directly induced by TH and simultaneously involved in various fundamental biological processes such as cell division, differentiation, apoptosis, morphogenesis, and cell-to-cell and cell-to-environment interactions (30). Although cell-to-cell interaction, some proteins, and genes involved in larval-to-adult muscle replacement have been reported (2, 30), TH-directed target genes and the global pattern of gene expression underlying this remodeling have yet to be characterized. The analysis of temporally changing transcription profiles after TH treatment is helpful for understanding the regulation of gene expression on a genome-wide scale in dorsal muscle remodeling and will provide a great value for developmental and endocrinology research. In this study, after TH, CHX, and TH_CHX treatment, we investigated the gene regulation profiles and identified TH direct target genes underlying the dorsal muscle remodeling during metamorphosis in *M. fissipes*.

This study offers the full-length transcriptome resource for *M. fissipes* using SMRT sequencing corrected with high accuracy and short Illumina reads. The mean length and N50 of full-length transcripts from this study, 2,222 and 2,299 bp, respectively, were much longer than those of *de novo* assembled transcripts (1,049 bp and 1,539 bp) and previous SMRT sequencing studies in the same species (22). Furthermore, higher percentages of transcript annotation are found in our SMRT sequencing analyses. Therefore, SMRT sequencing analysis in our study is useful and effective for acquiring reliable full-length transcripts of *M. fissipes*. A total of 16,234 lncRNAs were first predicted by all four methods in *M. fissipes*. Of 2,265 first predicted TFs, zf-C2H2 TFs were the most abundant, which also represents the largest class of putative human transcription factors (31). In animals, zf-C2H2 TFs can participate in tumors, cancers, and related gene regulation (32). The potential functions of these lncRNAs and TFs in *M. fissipes* need further study. Overall, this study improved the *M. fissipes* transcript characterization and annotation, and it will provide a valuable resource for future studies of candidate genes in anuran metamorphosis.

Making use of the transcriptome in *M. fissipes* dorsal muscle, we identified 1,245 DETs induced by TH, which should be a valuable resource for studying gene regulation and function during anuran muscle development and apoptosis. More dramatic changes in gene expression profile in CHX *vs.* control and TH_CHX *vs.* control were observed (**Figure 1**). CHX is a popular protein synthesis inhibitor in eukaryotes, which inhibits translation elongation by binding to the E-site of the 60S ribosomal unit and interfering with deacetylated tRNA (33). CHX induces rapid transcriptional upregulation of hundreds of genes involved in ribosome biogenesis (34), which are enriched in CHX *vs.* control and TH_CHX *vs.* control (**Figure S2**). Expectedly, CHX has altered thousands of downstream genes expression and led more quickly to the tadpoles’ death (in 1–2 days; data not shown).

Functional classification analysis using significantly detected DETs successfully revealed the associated function of the TH-modulated genes in dorsal muscle remodeling. GO enrichment analysis of DETs between the control and TH groups revealed that many DETs were involved and closely related to the cell division process, including biological process: protein import, protein location, protein activation, nuclear import, and transcription factor binding. In contrast, THs act *via* the TRs, which are primarily localized in the cellular nucleus. Nuclear import, which is crucial for the normal functions of TR, has enriched dorsal muscle after TH exposure (35).

In agreement with the GO analysis after TH treatment, the KEGG pathway related to cell proliferation, including protein export, nucleotide excision repair, base excision repair, nucleotide excision repair, protein processing in the endoplasmic reticulum, cell cycle, DNA replication, purine, and pyrimidine metabolism for DNA synthesis, was enriched. TH can influence DNA replication and cell cycle by activation of *CDK2*, *MCM2*, *MCM3*, *RFC5*, *TGFB*, *RNASEH2B*, and *CDKN1A* (11, 36, 37). Proteins were translated by ribosomes outside the endoplasmic reticulum (ER) and then modified post-translationally and folded in the ER (38). DETs such as *HSPA5*, *DNAJB*, and *DNAJC* in protein processing in endoplasmic reticulum pathway have been also reported in *X. laevis* during TH-modulated metamorphosis (11). Otherwise, apoptosis process-related pathways (including apoptosis-multiple species, p53 signaling pathway, and Nod-like receptor signaling pathway) were also enriched in our study. Apoptotic cell death regulated by TH has been reported in humans, amphibians, and insects (22, 39, 40). All DETs encoding MMPs and caspases (CASPs) were coordinately upregulated after TH was induced in muscle. The CASPs are a family of cysteine proteases that are known to regulate apoptotic signaling during metamorphosis (3, 4), while MMPs are a family of extracellular proteinases that have been shown to be important players for apoptosis in amphibian metamorphosis (22). p53 pathway plays an important role in regulating apoptosis; our data indicated that p53 is involved in muscle apoptosis during metamorphosis. In contrast, *Bcl-2*, *BIRC2*, and *BIRC3* involved in cell apoptosis were downregulated after TH treatment. *Bcl-2* constitutively suppresses p53-dependent apoptosis, while BIRC2 and BIRC3 are anti-apoptosis proteins that have the ability to regulate and inhibit the apoptosis process (41). Cell death and high cell proliferation activity detected after TH treatment of dorsal muscle proved that the “replacement model” is also detected in *M. fissipes*, which is the same as *Xenopus* (19). Furthermore, KEGG enrichment analysis also identifies significant changes in arachidonic acid metabolism and glycerophospholipid metabolism pathways, which is unsurprising because TH is a well-known regulator of lipid metabolism and arachidonic acid metabolites (42). Furthermore, cell apoptosis and cell proliferation were also detected in *X. laevis* intestine remodeling (11).

The DNA replication category and pathway were enriched after TH treatment, which were not enriched in TH_CHX-regulated gene. Interestingly, this was also found in *X. laevis* treated by TH and TH_CHX (11). These results indicated that the cell cycle process was induced by TH and TH_CHX; some later response genes, sensitive to CHX, are required for the cells to progress to DNA replication (11).

TH action is primarily mediated through TRs, which bind to TRE of direct response genes (11). Furthermore, CHX, which inhibits protein synthesis at the translation level, has been widely used for identifying the direct target gene of the nuclear receptor family, which constitutes an important group of TFs that control critical regulatory events in key developmental processes, homeostasis maintenance, and medically important diseases, such as TR and estrogen receptor (11, 43). DETs detected by TH exposure included both direct response and late response genes, and late response genes are sensitive to CHX. For the CHX and TH_CHX groups, tadpoles were treated with CHX 1 h earlier than TH, and DETs detected between the TH_CHX group and CHX group likely include CHX-resistant TH response genes and some CHX-affected genes (11). A total of 39 upregulated genes and 6 downregulated genes overlap between TH *vs.* control and TH_CHX *vs.* CHX, and these two comparisons identified the TH direct response gene. Of these potential TH directly regulated genes, *TRβ* and *TH/BZIP* were the well-known direct target genes in *X. laevis* and *X. tropicalis* during metamorphosis (44, 45). In addition to the direct target gene identified in amphibians, *KLF9* was verified to be directly regulated by TH in a TR-dependent manner in hematopoiesis and metamorphosis (46, 47), while *Matrix Gla protein* (*MGP*) gene is a target of TH in vascular smooth muscle cells *in vitro* and *in vivo*, which are associated with vascular calcification (48). Compared to the direct TH response genes detected in *X. laevis*, some genes are the same, such as *TRβ*, *TH/BZIP*, *SOX4*, and *KLF9* (11), which indicated that these four genes are TH direct response genes in amphibians. *TRβ*, *TH/BZIP*, and *KLF9* were conserved for TH regulated in vertebrates (44–47). *SOX4* is an essential developmental transcription factor that regulates stemness, differentiation, progenitor proliferation, and cancer cell proliferation (49). Less direct TH response genes were identified in our study because we just detected potential genes in muscle remodeling, not the whole body. Furthermore, we used more stringent standards to reduce the false-positive rate of the direct TH potential gene. Then, we used qPCR to analyze 12 newly potential TH target genes in *M. fissipes*, all consistent with the data obtained from RNA-seq. The expression level of these 45 direct response genes changed dramatically and rapidly after TH exposure, so these genes could also be potential biomarkers to assess the effects of environmental contaminants on TH signaling and endocrine. Frog tadpoles are very sensitive to environmental substances because of their habitat and the complex processes of metamorphosis regulated by the endocrine system, mainly thyroid hormones (50). Therefore, the larval *M. fissipes* could be used for screening and testing potential endocrine disrupters using these sensitive genes.

For TH directly regulated genes, binding sites are often formed by a direct repeat of two AGGTCA hexamers separated by four bases (11). By bioinformatics analysis, AGGTCAnnTnAGGTCA is predicted as the optimal target sequence of endogenous target genes for TR/RXR heterodimers in *M. fissipes*. Not only the consensus sequence AGGTCA but also the 4-bp spacer sequence with “T” abundant at the third position is consistent with previous findings in *Xenopus* (11).

Through the PacBio SMRT sequencing platform, a large collection of full-length transcripts was obtained. The number and mean length of the unigenes, as well as the number of complete open reading frames (ORFs), from SMRT sequencing were much better than those from Illumina sequencing. This study provides a foundation for elucidating the molecular map for the dorsal muscle remodeling in *M. fissipes*. The different transcriptional signatures linked to cell differentiation and apoptosis have been identified. Our study is the first to find the TH target genes in *M. fissipes*, especially in dorsal muscle remodeling. Future investigations on the function and regulation of these genes and pathways should help to elucidate the mechanisms governing amphibian dorsal muscle development and apoptosis.

## Data availability statement

The datasets presented in this study can be found in online repositories. The names of the repository/repositories and accession number(s) can be found below: https://ngdc.cncb.ac.cn/gsa/, CRA008592.

## Ethics statement

The animal study was reviewed and approved by the Experimental Animal Use Ethics Committee of the Chengdu Institute of Biology, Chinese Academy of Sciences.

## Author contributions

LL, ZG, and JJ conceived and designed the study. LL performed the sample collection and molecular experiments, analyzed the data, and wrote the manuscript. QL and XZ helped with qPCR. XW and QC assisted with the bioinformatics analysis. All authors contributed to the article and approved the submitted version.

## Supplementary material

Supplementary Figure 1
**(A)** Venn diagram of the number of candidate lncRNAs predicted using CPC, CNCI, PLEK and Pfam analysis, respectively. Overlapping areas indicate the number of lncRNAs identified by the several tools, while un-overlapping areas indicate the number of lncRNAs identified only by the single tool. **(B)** Transcription factor (TF) families identified in this study.

Supplementary Figure 2 Heatmap of all DETs in four groups (Control group, CHX treatment group, TH treatment group, and TH_CHX treatment group) based on RNA-seq. The intensity of color indicates relative expression levels. Red indicates that the gene is highly expressed in the group, whereas green indicates low expressed.

Supplementary Figure 3 The characteristics of GO terms significantly enriched between Control group and TH_CHX treatment group.

Supplementary Figure 4 The characteristics of KEGG pathways significantly enriched between Control group and TH_CHX treatment group.
