# Supplementary figures and images for: Identification of thyroid hormone response genes in the remodeling of dorsal muscle during Microhyla fissipes metamorphosis
Source: Front Endocrinol (Lausanne). 2023 Feb 3;14:1099130. doi: 10.3389/fendo.2023.1099130 (PMC9937655; doi:10.3389/fendo.2023.1099130)

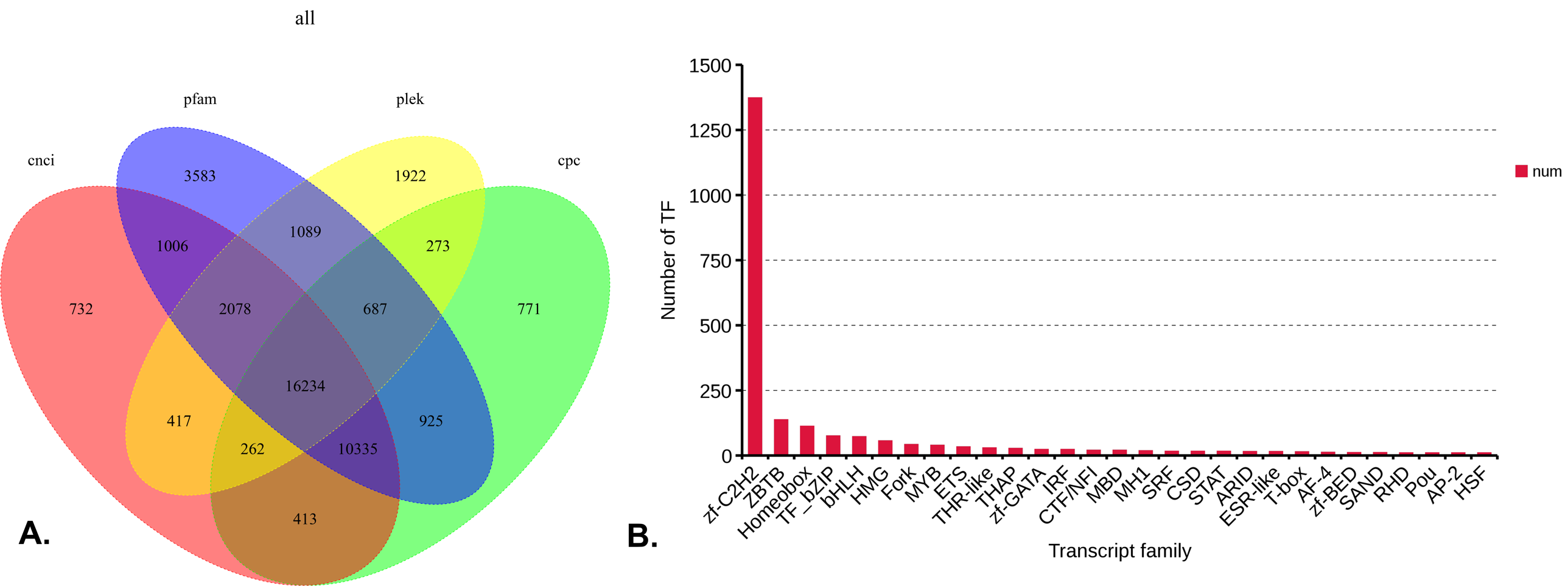

Supplement: Supplementary Figure 1 — (A) Venn diagram of the number of candidate lncRNAs predicted using CPC, CNCI, PLEK and Pfam analysis, respectively. Overlapping areas indicate the number of lncRNAs identified by the several tools, while un-overlapping areas indicate the number of lncRNAs identified only by the single tool. (B) Transcription factor (TF) families identified in this study. [file Image_1.tif]

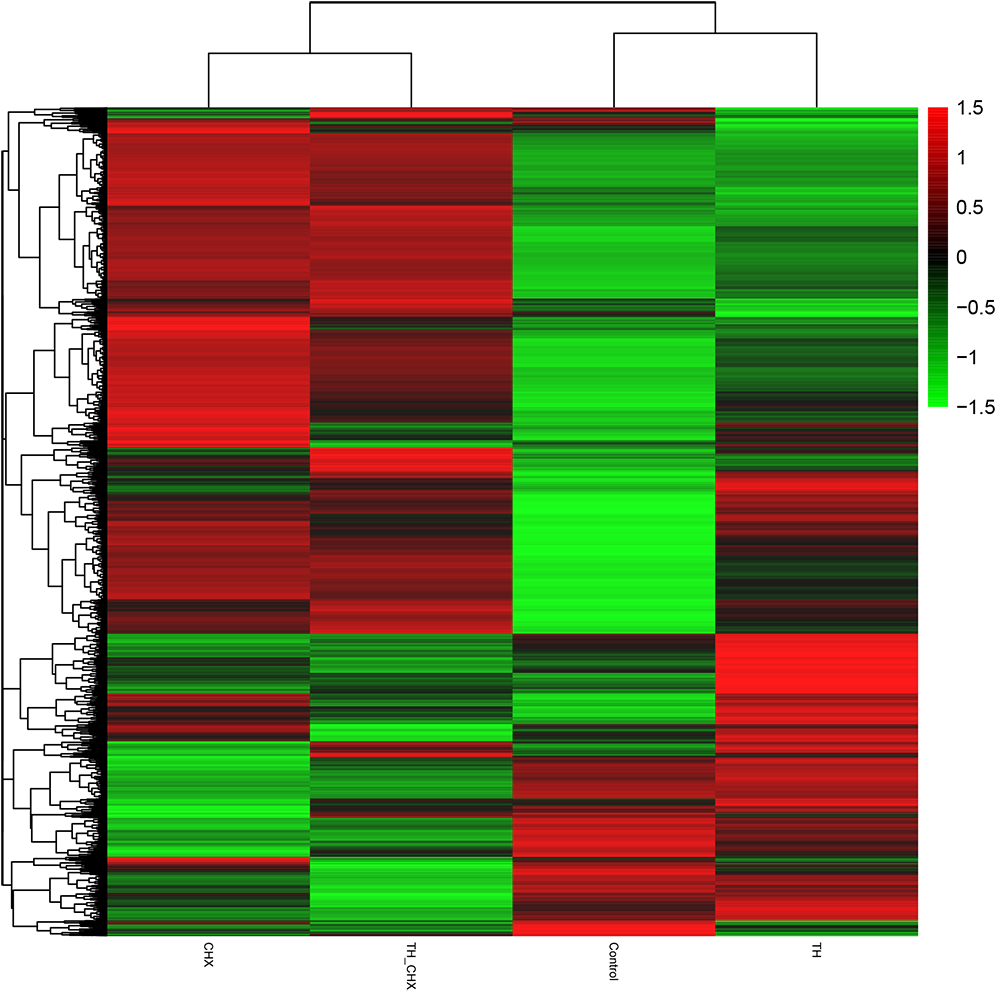

Supplement: Supplementary Figure 2 — Heatmap of all DETs in four groups (Control group, CHX treatment group, TH treatment group, and TH_CHX treatment group) based on RNA-seq. The intensity of color indicates relative expression levels. Red indicates that the gene is highly expressed in the group, whereas green indicates low expressed. [file Image_2.tif]

## TH\_CHXvsControl (GO)

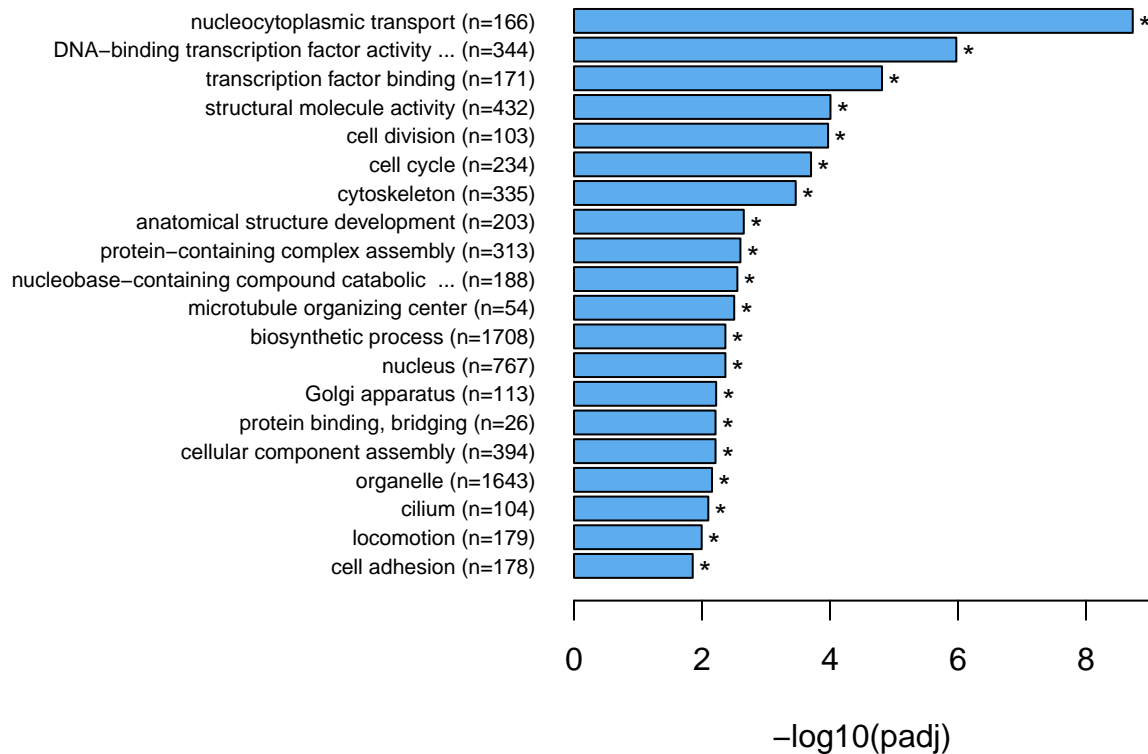

Supplement: Supplementary Figure 3 — The characteristics of GO terms significantly enriched between Control group and TH_CHX treatment group. [file DataSheet_1.pdf]

# TH\_CHXvsControl

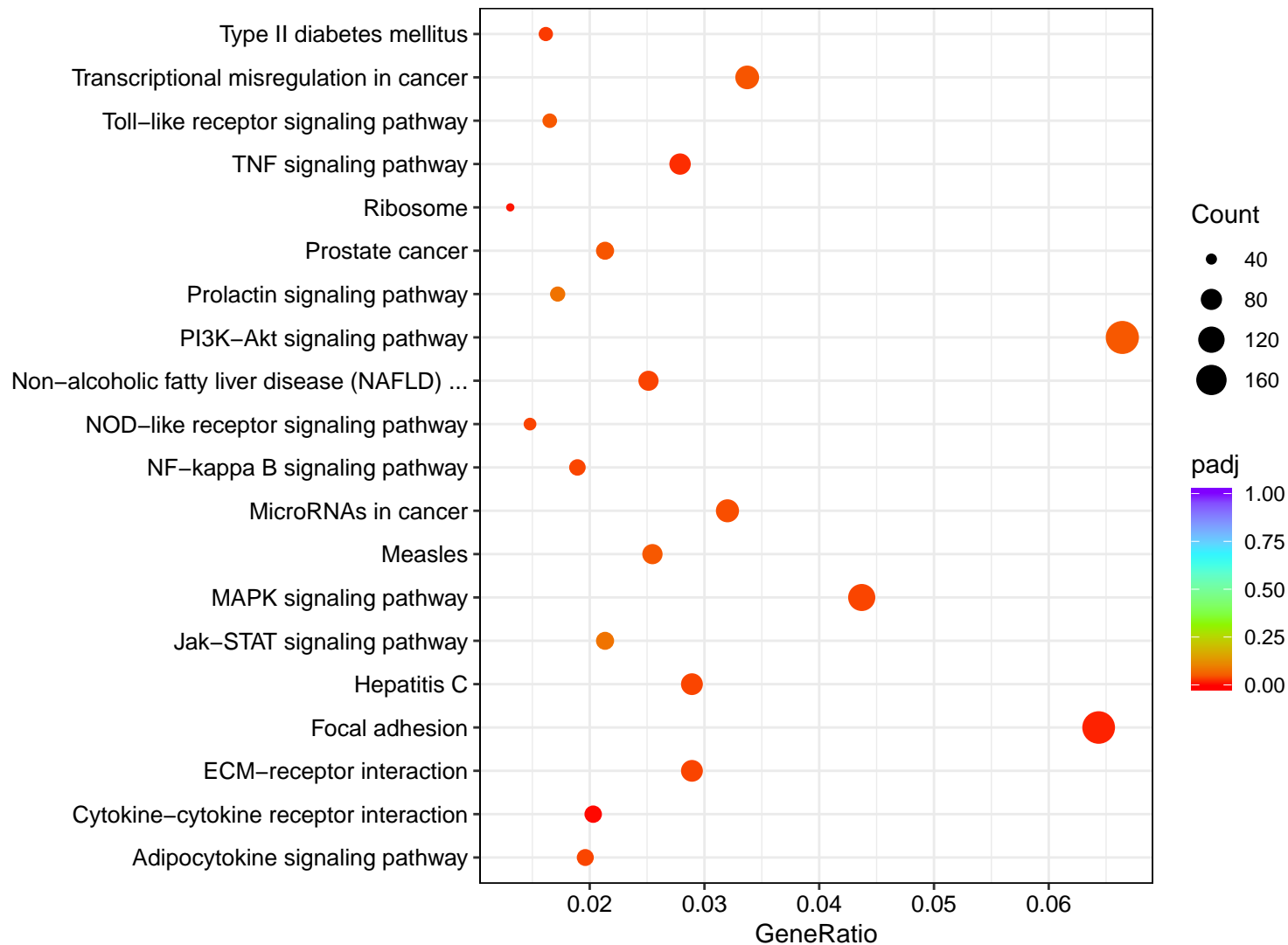

Supplement: Supplementary Figure 4 — The characteristics of KEGG pathways significantly enriched between Control group and TH_CHX treatment group. [file DataSheet_2.pdf]
